# Bacterial Cellulose—Adaptation of a Nature-Identical Material to the Needs of Advanced Chronic Wound Care

**DOI:** 10.3390/ph15060683

**Published:** 2022-05-30

**Authors:** Paul Zahel, Uwe Beekmann, Thomas Eberlein, Michael Schmitz, Oliver Werz, Dana Kralisch

**Affiliations:** 1 JeNaCell GmbH—An Evonik Company, 07745 Jena, Germany; paul.zahel@evonik.com (P.Z.); uwe.beekmann@evonik.com (U.B.); 2 Department of Pharmaceutical/Medicinal Chemistry, Institute of Pharmacy, Friedrich Schiller University Jena, 07743 Jena, Germany; oliver.werz@uni-jena.de; 3 Akademie-ZWM AG, 8424 Embrach, Switzerland; thomaseberlein@hotmail.com; 4 MCS Medical Consulting, 56414 Oberahr, Germany; schmitzmichael@mcsoberahr.onmicrosoft.com; 5 Evonik Operations GmbH, 45128 Essen, Germany

**Keywords:** bacterial cellulose, carbohydrate polymer, advanced wound care, chronic wounds, clinical data, exudate management, moisture vapor transmission rate

## Abstract

Modern wound treatment calls for hydroactive dressings. Among the variety of materials that have entered the field of wound care in recent years, the carbohydrate polymer bacterial cellulose (BC) represents one of the most promising candidates as the biomaterial features a high moisture-loading and donation capacity, mechanical stability, moldability, and breathability. Although BC has already gained increasing relevance in the treatment of burn wounds, its potential and clinical performance for “chronic wound” indications have not yet been sufficiently investigated. This article focuses on experimental and clinical data regarding the application of BC within the indications of chronic, non-healing wounds, especially venous and diabetic ulcers. A recent clinical observation study in a chronic wound setting clearly demonstrated its wound-cleansing properties and ability to induce healing in stalling wounds. Furthermore, the material parameters of BC dressings obtained through the static cultivation of *Komagataeibacter xylinus* were investigated for the first time in standardized tests and compared to various advanced wound-care products. Surprisingly, a free swell absorptive capacity of a BC dressing variant containing 97% moisture was found, which was higher than that of alginate or even hydrofiber dressings. We hypothesize that the fine-structured, open porous network and the resulting capillary forces are among the main reasons for this unexpected result.

## 1. Introduction

Chronic wounds represent an enormous burden for patients and caregivers and have an immense economic impact on society [1,2]. Studies estimate that between 1 and 4% of total healthcare expenditure in developed countries is spent on chronic wound treatment and related interventions [1,3]. One important prerequisite for successful wound management is the selection of a suitable wound dressing [4,5].

Wounds can be described as disruptions in the epithelial integrity of the skin due to physiochemical or thermal damage or medical pathology. Consequently, essential skin barrier functions, such as thermal insulation and protection against pathogens or external mechanical damage, are impaired [6,7,8]. Although acute wounds usually progress through the four temporally and spatially overlapping stages of hemostasis, inflammation, proliferation, and remodeling, and close within 8–12 weeks, chronic wounds typically remain in the inflammatory stage, exceeding this period and may be associated with heavy exudation, pain, or infections, and can lead to sepsis or amputations [8,9,10,11].

Although the causes of chronic wounds are highly diverse, the majority of cases share similar underlying processes and can be classified into the categories of pressure ulcers, vascular ulcers (venous, arterial, or mixed leg ulcers) and diabetic ulcers [11,12]. Pressure ulcers often occur in patients with compromised mobility, in which case the tissue is subjected to unrelieved pressure that exceeds the capillary perfusion, leading to ischemia, hypoxia, and thus tissue necrosis [11,12,13]. In venous leg ulcers (VLUs), damaged or incompetent vein valves lead to venous stasis, hypertension, and oedema [13]. The increased pressure exceeds capillary perfusion pressure and blood vessel permeability resulting in macromolecules leaking into the perivascular space. Following ischemia, oedema and hypoxic conditions impede healing of minor trauma and accelerate skin breakdown, which is associated with inflammation and bacterial invasion. Arterial ulcers are much rarer than venous ulcers and result from arterial insufficiency due to atherosclerosis or arterial embolism in the extremities. A narrowed arterial vessel lumen and ischemia of the tissue are associated with hypoxia, through which healing of minor trauma is severely affected and tissue breakdown is accelerated. Lastly, diabetic (foot) ulcers differ from other chronic wound types on account of their specific pathophysiology. Diabetic peripheral polyneuropathy manifests in sensory loss, senso-motorical separation, atrophic structural changes, and thus an increased risk of ulceration from repeated mechanical stress. Impaired perfusion and metabolic derangements further disrupt wound healing and contribute to the chronification of diabetic ulcers [11,12,13,14].

Although treatment of the underlying diseases and causes is critical to successful wound management, the principles and advantages of a moist healing milieu, which were first studied by Winter in 1962 [15], are meanwhile widely accepted and backed by evidence [16,17]. A moist wound environment promotes autolytic debridement [18], the proliferation of keratinocytes and fibroblasts [19,20], and collagen synthesis [21], which leads to less pain, reduced scar formation, and faster wound closure [16,17,22].

In order to benefit from these findings in clinical praxis, a plethora of different materials for advanced (chronic) wound care have been developed over the past decades. Dependent on the wound’s exudate level, one can choose from highly absorbant polyurethane foam dressings, hydrogel-forming carbohydrate polymers such as alginate and carboxymethylcellulose dressings, occlusive film and hydrocolloid dressings, or even moisture-donating products such as hydrogels and bacterial cellulose dressings [23,24]. Furthermore, many promising dressing developments based on techniques such as electrospinning or 3D bioprinting have been reported in recent years. These dressings show broad applicability and feature advantageous properties such as the controlled release of incorporated drugs or antibacterial activity [25,26]. Today, they are primarily used in regenerative medicine and tissue-engineering techniques [27].

Although there are some dressings that should be used primarily for special indications such as burns, the selection of an appropriate dressing is based less on the classification of the wound as chronic or acute and more on its characteristics such as tissue perfusion, exudate level, the occurrence of necrosis and infection, or localization of the wound [4]. Above all, ensuring a moist wound environment, protection against microorganisms, and thermal insulation are of high importance in both wound classifications [28]. Thus, the challenge of finding a material that sufficiently addresses all these properties and at the same time provides a moist wound environment has not yet been adequately overcome. Bacterial cellulose, however, may be a very promising candidate to fulfill these requirements.

The usage of polysaccharide materials has a long history in the field of wound care; from oil-soaked linen strips over traditional cotton gauze to advanced wound-care products [29]. In particular, semi-synthetic cellulose derivatives such as carboxymethylcellulose possess numerous advantageous properties and are nowadays of great value in advanced wound care [30]. However, carbohydrate-biopolymer bacterial cellulose (BC) has recently attracted greater attention as the subject of numerous works. In contrast to plant-derived cellulose, BC is biosynthesized by various bacteria strains from glucose monomers that form a three-dimensional network of interconnected nanostructured cellulose fibers [31,32]. It can be produced on a large scale by controlled fermentation [33]. In this process, planar hydrogel fleeces are formed on the surface between the nutrient medium and the air. Those dimensionally stable BC hydrogel membranes can be applied as wet wound dressings directly after purification and sterilization without further processing [34]. However, since the selected bacteria strain, as well as the biotechnological production process, can influence the key characteristics and material properties, it is important to independently investigate the suitability of each strain for the intended application [35]. For the strain *Komagataeibacter xylinus* DSM 14666 (see Methods section), an extensive basis of experimental data for the mentioned properties is already available from previous works. Dressings produced using this strain show, for example, a well-suited moisture vapor transmission rate (MVTR) of around 3000 g/m^2^/24 h [36,37] when measured in contact with vapor. Excellent biocompatibility of the unmodified material could be shown in vitro for RAW264.7 (murine macrophage-like cells) and THP-1 (human monocytic/macrophage-like cells) in standard MTT assay [36,37] as well as for HaCaT keratinocytes in luminometric ATP assay [38]. Furthermore, ex-ovo biocompatibility using a shell-less hen’s egg test on chick area vasculosa (HET-CAV) was once more confirmed, and in vitro wound healing in a HaCaT scratch assay showed no negative effects on cell monolayer closure [36,37]. All moist BC wound dressings have in common high biocompatibility, water-holding capacity, conformability, vapor permeability, and mechanical stability while providing a physical barrier against bacteria and other pathogens. Compared to many alternative products, BC also stands out for its high water-holding capacity, which helps to create a moist wound environment. This is due to the microscopic structure of the material: the interconnected fibers with a diameter of less than 100 nm result in a remarkable surface area and enclose large amounts of water, which stabilizes the cellulose network via hydrogen bonds [31,39]. With regard to these characteristics, BC dressings have been shown in several clinical observations to effectively relieve pain, absorb and retain exudate, provide an optimal moist wound environment, diminish infection rates, and last but not least, hasten re-epithelization and shorten wound healing. [34,40,41,42,43,44]. An overview of previously published clinical data as well as key findings can be found in the Supplementary Materials, Tables S1 and S2.

Clinical data published so far focused on clinical experiences with BC wound dressings in the treatment of burn wounds and chronic wounds alike without taking into account the high variability of the material, which allows an adaption of material properties on the wound type. Previous work furthermore focused on investigating the underlying mechanisms on a cellular level. Although the properties of BC that promote wound healing cannot yet be fully explained, some specific mechanisms have been observed in the past. After Sanchavanakit et al. first demonstrated that BC dressings support the growth, spreading, and migration of human keratinocytes in vitro [45], other groups observed accelerated angiogenesis, tissue regeneration, and collagen expression in vivo [46,47]. The high water content of the dressings has been shown to induce a local cooling effect in ex vivo burn wounds, reducing intradermal temperature and thermal damage [48]. In recent years, it has been shown that an acidic pH value is favorable for fast and successful wound healing, e.g., chronic wounds show an alkaline pH [49]. BC dressings that had an acidic pH were associated with faster wound healing than neutral or alkaline dressings, which is a promising finding for future clinical applications [50].

However, in addition to the positive effects of the unmodified material, many studies have taken the development one step further and demonstrated the strong potential of the carbohydrate-based material as a drug carrier to treat chronic wounds. To give an example, Hoff et al. showed how the controlled release of α-13’-carboxychromanol, a long-chain vitamin E metabolite, promoted wound healing and closure in a diabetic mouse model [51].

What is still missing is the thorough analysis of the material properties of BC-based wound dressings and the differences as well as similarities with respect to other advanced wound-dressing materials. Consequently, there is a lack of experience as to which parameters must be adjusted to adapt and optimize a BC-based wound dressing to the individual requirements of certain indications or stages of wound healing.

To gain further clinical experience and identify the potential for the optimization of the material with regard to chronic wounds, a post-market clinical follow-up study featuring epicite^hydro^, a BC wound dressing already widely used in burn treatment, was carried out. The clinical observation was accompanied by comparative analyses of the material properties in standardized tests and adapting bacterial cellulose dressings to the specific requirements identified in the clinical and in vitro tests. The results of the combined studies could provide further important information for the general understanding of the unique aspects of BC in wound healing. Moreover, the study aims to shed light on how this biomaterial can be adapted to the specific requirements of treating different kinds of wounds.

## 2. Results and Discussion

### 2.1. Post-Market Clinical Follow-Up Study

The hydroactive BC wound dressing epicite^hydro^ (BC_A) has already been proven to perform very well in the treatment of burn wounds [52,53,54,55]. In order to investigate its clinical performance in chronic wound treatment, a PMCF observation study with 44 patients suffering from venous leg ulcers, mixed leg ulcers, and diabetic foot syndrome was carried out.

**Figure 1:**
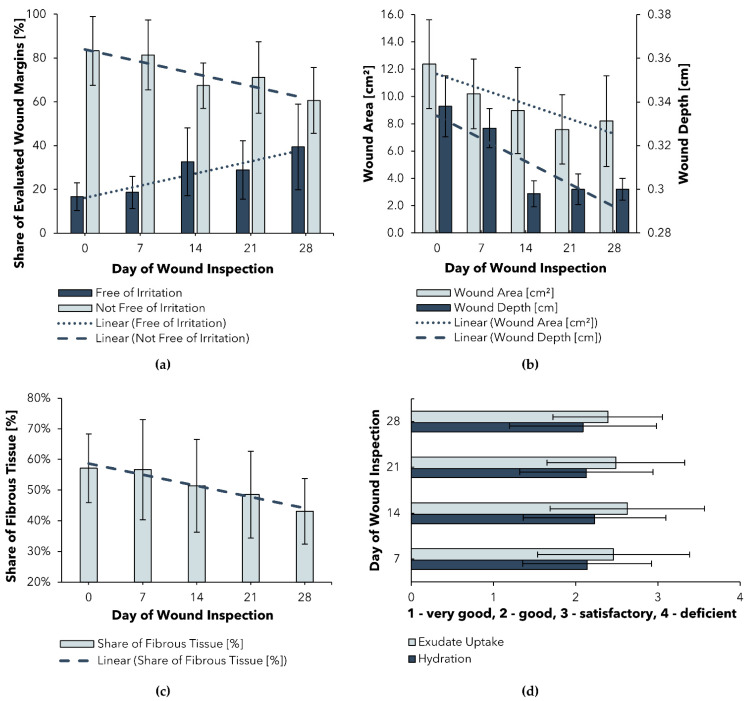
Selected results of a Post-Market Clinical Follow-up (PMCF) study of BC_A: (**a**) Progression of wound area and depth; (**b**) Progression of wound margin irritation; (**c**) Progression of fibrous tissue share; (**d**) Assessment of exudate management; time points of wound inspections ± 3 days.

Selected results with respect to (a) wound area and wound-depth progression, (b) share of non-irritated wound margins, (c) share of fibrous tissue, as well as (d) exudate update and hydration are shown in Figure 1.

Figure 1a shows a significant (*p* < 0.006) reduction in the mean wound size and depth over the study period of 28 days. Both results can be explained, at least in part, by the ability of BC_A to create a moist environment that promotes the healing of even stagnating chronic wounds, proving that this ability of BC_A is not limited to burn wounds. For BC-based wound dressings in general, these results tie in well with previous clinical trials, wherein the suitability for chronic wounds was demonstrated (Supplementary Materials, Table S1). Another promising result is the consistent and significant (*p* = 0.001) reduction in fibrous tissue (c), which can be explained by the high moisture content of the wound dressing. The associated cleansing effect could be attributed to a softening of the plaques and therefore facilitation of autolytic debridement. A similar conclusion was reached by Alvarez et al. in a randomized clinical trial analyzing a wet BC dressing for the treatment of venous leg ulcers [56]. Future works should compare the wound-cleansing efficiency achieved using a dry dressing as a control group.

Since an excess of moisture at the dressing–wound interface can lead to macerations of the periwound skin, the condition of the wound margins needs to be carefully observed [57]. In the PMCF study, it was shown that the share of wound margins free of irritation significantly increased during the observed trial period (Figure 1b). These results suggest that BC_A may even have a desirable soothing effect on irritated periwound skin.

Although the aforementioned results indicate high suitability of BC_A in the treatment of chronic wounds, a survey of participating users revealed that exudate uptake was rated only as “good” to “satisfactory” and wound hydration as “good”. Since exudate management plays a crucial role in successful wound treatment [24,57] and is one important factor in dressing selection, an enhancement of the exudate-handling properties of BC_A is desirable to optimize the dressing for chronic wound treatment.

In the following sections, the material properties of BC wound dressings compared to other advanced wound dressings will be discussed to provide recommendations for the adaption of the dressing for further use in exuding wound environments based on experimental evidence. The work focused on exudate-handling properties under the free-swell condition as well as compression, moisture donation capacity, and the permeability measured using the MVTR. To increase the capability of the experimental BC to absorb wound exudate, certain cultivation parameters were varied, and the dressings underwent a partial dehydration process. An overview of all evaluated parameters, tests, and results is shown in Section 2.7.

### 2.2. Solid Content

**Figure 2:**
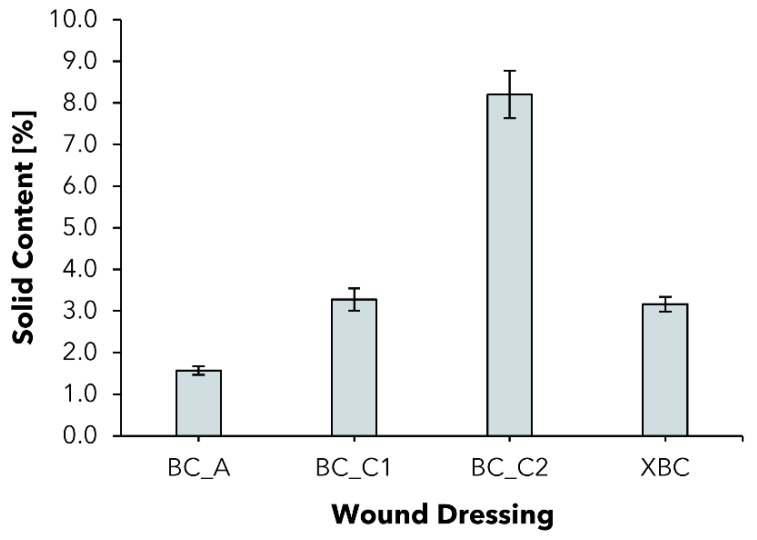
Solid content of wet bacterial cellulose wound dressings (mean ± SD; *n* = 8).

To characterize the commercial and experimental BC dressings, first regarding their hydration level, the solid content (SC) of the experimentally produced wet BC wound dressings (BC_C1; BC_C2) and commercial wet BC dressings epicite^hydro^ (BC_A) and Suprasorb X (XBC) was determined (Figure 2). The solid content of a specific BC dressing describes the proportion of BC in the total mass of the sample. The parameter is of interest since moist BC wound dressings can be described as hydrogel bodies consisting of a varying amount of water and an interconnected 3D network of thin cellulose fibers [31,32]. All other commercial wound dressings investigated were excluded from this experiment since they are dry.

BC_A represents a moist dressing with an SC of 1.57 ± 0.10%, which is in accordance with former works [36]. The value was increased by more than two, resp. five times to 3.27 ± 0.27% for BC_C1 and 8.20 ± 0.57% for BC_C2. The commercial product XBC is comparable to BC_C1 in terms of the SC. Since the dressings tend to reswell to their original water resp. solid content when in contact with excess liquid such as wound exudate, the increased solid content represents the starting point for an improved exudate absorption.

### 2.3. Free Swell Absorptive Capacity

**Figure 3:**
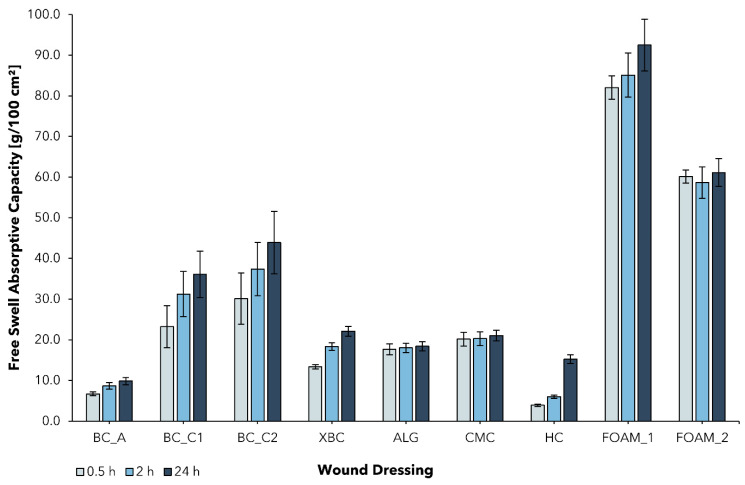
Free swell absorptive capacity (AC) of bacterial cellulose wound dressings and alternative wound care products (mean ± SD; *n* = 5). The fluid uptake was measured at three successive time points (AC_0.5_; AC_2_; AC_24_) in the same experimental setting for epicite^hydro^ (BC_A); Suprasorb^®^ X (XBC); Suprasorb^®^ A (ALG); Aquacel^®^ Extra™ (CMC); Hydrocoll^®^ (HC); ALLEVYN Gentle (FOAM_1); Mepilex^®^ (FOAM_2).

Although a moist healing environment is widely accepted as beneficial for successful wound healing and represents the standard for modern wound management, an excess of exudate can lead to wound complications and skin deterioration (e.g., irritation, maceration) [57,58]. In contrast to acute wounds, exudate in chronic wounds differs in its composition and has been shown to slow down cell proliferation, interfere with growth factor availability, and lead to successive degradation of the extracellular matrix [24,57]. To maintain an optimal moisture balance, a dressing must be able to absorb excess exudate and hydrate the wound at the same time. In order to compare the BC-based wound dressings, the free swell absorptive capacity according to EN 13726-1:2002 was determined for BC_A, BC_C1, BC_C2, XBC, and five different commercial wound dressings that represent a variety of commonly used options for the treatment of chronic wounds in Germany (Figure 3).

BC_A, typically used in the treatment of burns, showed a relatively low AC_0.5_ of 6.7 ± 0.5 g/100 cm^2^, which can be attributed to its high water content (>98%, see Figure 2). The cellulose network is already saturated with water and thus not well-suited for further fluid uptake. This property has proven to be beneficial in the treatment of burn wounds in multiple studies, mainly due to the dressing’s ability to provide a moist wound environment [52,53,55]. However, in the treatment of chronic wounds, exudate absorption is of higher importance. Never-dried BC dressings feature the ability to almost completely reswell after the mechanical removal of water. In this work, the combination of increasing the thickness of the initial cellulose layer together with the intensified removal of water aimed to improve its ability to absorb aqueous fluids such as wound exudate. The determined AC_0.5_ of the produced dressings BC_C1 and BC_C2 was found to be more than 3, resp. 4 times higher compared to BC_A and exceeded the AC_0.5_ of XBC by 2, resp. 3 times. This finding suggests that increased solid content leads to the higher absorptive capacity of BC-based wound dressings, which could therefore be more suitable for the therapy of medium to highly exuding wounds.

Hydrofiber and alginate dressings form gels instantly, resp. over a period of several hours when in contact with exudate, and are used for highly exuding, deep wounds [23]. In the performed test, ALG and CMC were within a range of AC_0.5_ = 15–20 g/100 cm^2^, which is in line with the literature [59,60]. They exceeded BC_A in terms of their absorbency but fell short of BC_C1 and BC_C2. The tested foam dressings were observed to absorb the most exudate of all dressing classes investigated and feature an AC_0.5_ of ~60–85 g/100 cm^2^, which compares well with previously published data on the AC of commercial foam dressings and can be explained by the open structure of the foam network [61].

It was further observed that all BC-based wound dressings were not saturated within the test period of 0.5 h and continued to absorb exudate beyond the test duration of 2 h, whereas the other tested dressings showed little difference between AC_0.5_ and AC_24_.

### 2.4. Absorptive Capacity under Pressure

**Figure 4:**
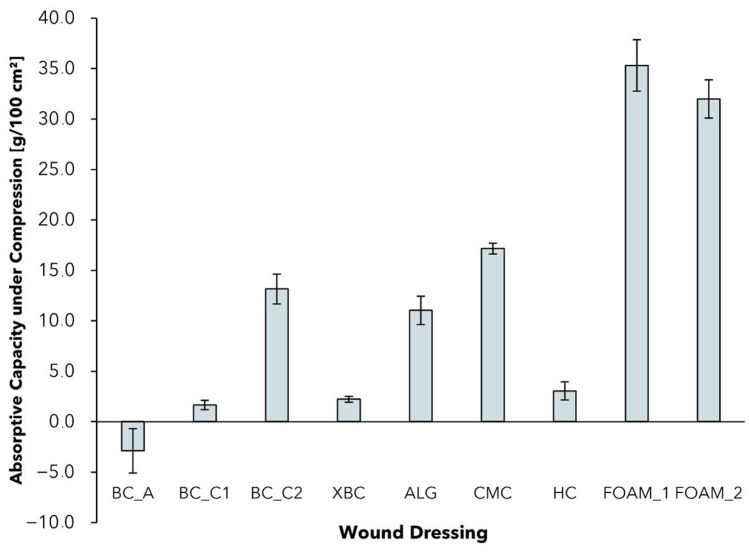
Absorptive capacity of different wound dressings after 0.5 h and under compression of 40 mmHg (mean ± SD; *n* = 5).

Although the standardized measurement of the free swell absorptive capacity is a good indication for the comparison of different wound dressings, it is only partially representative for specific applications [62]. In particular for patients with chronic wounds due to oedema or venous leg ulcers, compression therapy is indicated and represents a well-established standard [63]. The applied pressure can lead to a reduction in venous pooling volume, and an improvement in muscle pump function and venous blood flow [64]. Effects such as increased ulcer healing rates and reduced healing duration under compressive bandages are supported with evidence [64]. The successful use of a wound dressing to treat chronic wounds from, e.g., venous ulcers, therefore, also depends on its capability to absorb and retain exudate under the influence of mechanical pressure. To analyze the absorption behavior in that specific case, a method based on prEN 13726 was selected with the samples subjected to a pressure of 40 mmHg corresponding to compression class 3 (CCL3, Figure 4) [65].

In comparison to free swell AC (Figure 3), all wound dressings exhibited reduced AC_0.5_ values under the influence of pressure, with CMC dressings showing the lowest reduction of 16.7%. It could be observed that the materials react differently to the influence of mechanical pressure and vary in their ability to retain exudate. The AC_0.5_ of the tested foam dressings decreased by a range of AC_0.5_ = 30–40 g/100 cm^2^. The differences in AC reduction can be explained by the different mechanisms of water absorption and retention. Although foam and BC-based dressings feature an open structure with free swell absorptive capacity, dressings such as ALG and CMC form hydrogels with the absorbed exudate [13,66]. Despite the fact that the absorbed water in foam dressings can be considered as “free”, the fluid that is bound in a hydrogel can be retained better under compression [66].

Negative AC_0.5_ in the case of BC_A can be explained by the fact that the material is already saturated with moisture, which is partly pressed out of the dressing by mechanical force. Looking at the BC-based wound dressings, BC_C2 performed best in terms of absorptive capacity, whereas BC_C1 and XBC lost most of their absorptive capacity. The reduced AC, especially of BC_A, should therefore be considered when treating chronic wounds that require compression therapy. With regard to that application, foam and hydrofiber dressings perform well in particular, but dressings such as BC_C2 with an increased solid content also show promising results.

### 2.5. Moisture Vapor Transmission Rate

Since the physiological skin barrier is damaged in the case of a wound, the evaporation of water represents a key factor in the regulation of a wound’s moisture level. The loss of water from a wound site mainly takes place through absorption from the wound dressing and evaporation through and from the dressing. Therefore, the moisture vapor transmission rate (MVTR) of a chosen dressing is of fundamental importance in the exudate management of a wound [16,67]. To compare the evaporation of water through different wound dressings, the standardized Paddington cup method as described in EN 13726 was chosen.

**Figure 5:**
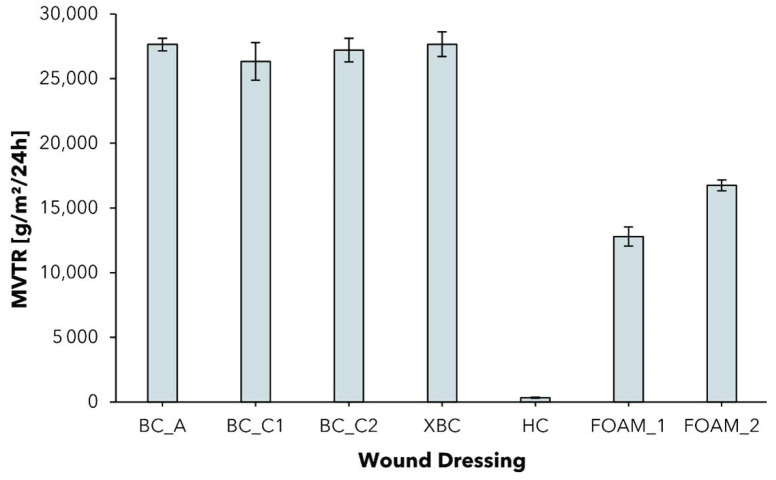
Moisture vapor transmission rate (MVTR) of different wound dressings determined with standard Paddington cup in contact with liquid method (mean ± SD; *n* = 5).

As described in the standard, MVTR data was determined for two different experimental settings: dressings in contact with vapor (Supplementary Materials, Figure S1) and dressings in contact with liquid (Figure 5).

Although the contact with liquid method is recommended because it simulates more closely the wound environment as the dressing is in direct contact with the exudate [68], some dressings (see ALG; CMC) do not feature waterproofness and can therefore only be analyzed by the contact with vapor method (see Supplementary Materials, Figure S1).

In previous works as well as in this test, it was observed that all dressings showed relatively high MVTR values when in contact with liquid [69]. The BC dressings exceed the MVTR of foam dressings, the latter exhibiting an MVTR range close to that of the BC dressings in contact with vapor. Furthermore, the increase in water permeability when in contact with liquid instead of vapor was found to be remarkably lower for the foam dressings. Since BC dressings featuring an MVTR of >25,000 g/m^2^/24 h when in contact with water exhibit lower absorptive capacity than foams, the mechanism of exudate removal is likely to be more pronounced on evaporation than on absorption. Similar results concerning the exceptional increase in water permeability of a bacterial cellulose wound dressing were published for the first time by Thomas [70]. However, they have never before been set in the context of the surprisingly high free swell absorptive capacity values. We hypothesize that the fine-structured, open-porous structure network of hydrophilic cellulose fibers supports moisture transport by capillary forces from the wound through the dressing to the surrounding air or the second dressing, facilitating a unique hydrobalance property.

In the case of the contact with vapor method, the MVTR values obtained were in a broad range. Hydrocolloid dressing (HC) showed the lowest MVTR of 98 ± 5 g/m^2^/24 h, and can, therefore, be categorized as an occlusive dressing (MVTR < 300 g/m^2^/24 h [5]). The low MVTR can be explained by their composition of an outer layer as being a polymeric film, which holds a layer of absorbent hydrocolloids such as sodium carboxymethyl cellulose and gelatine as well as adhesives and gelling agents [32,33]. Occlusive dressings are suited for use on low or no-exuding wounds to keep a moist wound environment, but can lead to periwound skin maceration and dressing leakage on wounds with higher levels of exudation and are not recommended to be used on infected wounds [5,28,71,72].

The MVTR in contact with vapor for the tested BC-based wound dressings was found to be in a range of 2697 ± 118 g/m^2^/24 h (BC_C1) to 4039 ± 230 g/m^2^/24 h (BC_A), which is in accordance with previously published data (MVTR~3000 g/m^2^/24 h) [36,37] and close to the ideal MVTR range reported for maintaining the optimal moisture content of a wound (2000–2500 g/m^2^/24 h, measured in contact with vapor) [73,74]. In the case of BC_A, the high MVTR is compensated by the intrinsic moisture of the dressing, which hydrates the wound and maintains a moist environment. Although the increased cellulose content and thickness of the produced dressings BC_C1 and BC_C2 clearly decreased the moisture vapor permeability, the MVTR was still found to be in the optimal range. With regard to the clinical application, this finding suggests that an increased absorptive capacity is not at the expense of an adequate permeability of moisture in vapor form.

### 2.6. Fluid Donation

Although the successful healing of many types of chronic wounds is likely to be affected by an excess of exudate, the moisture balance needs to be evaluated continuously. Whereas fibrous tissue and other plaque often need to be removed first by, e.g., an autolytic debridement before the healing process can be reactivated in a stagnating wound, the exudate level also changes as healing progresses to the later stages. In such cases, external hydration (e.g., via moist dressings) can ensure an optimal moisture level and thus successful wound closure [75].

Since most wound-dressing products used today in the treatment of chronic wounds come in a dry state to absorb an excess of exudate, the native hydration of BC-based wound dressings such as BC_A or XBC represents the rare property they share with only a few products, such as amorphous hydrogels or hydrogel dressings. To evaluate the influence of an increased solid content and to compare BC dressings in terms of their fluid donation (FD) capability to an artificial gelatin wound bed, a method based on EN 13726 was adapted.

**Figure 6:**
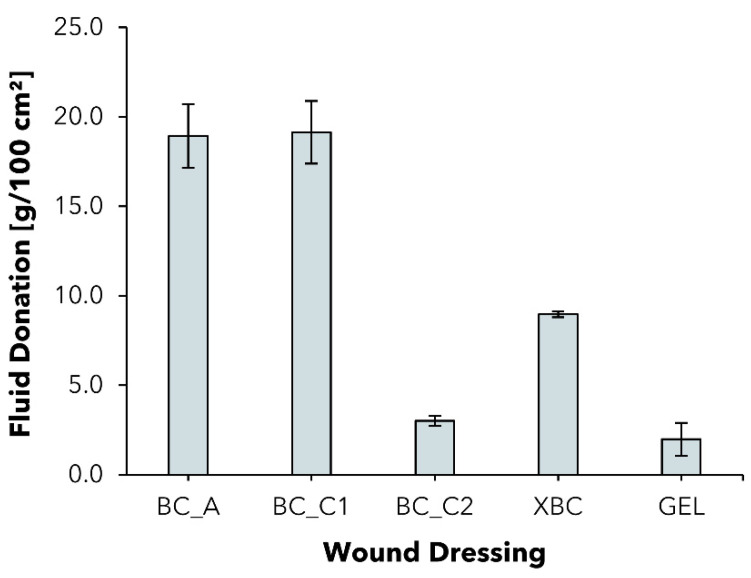
Fluid donation per area of five different moisture-donating wound dressings to a gelatin substrate after 48 h (mean ± SD; *n* = 5).

In a comparison of the tested moisture-donating commercial wound dressings, BC_A showed the highest FD of 18.9 ± 1.8 g/100 cm^2^, whereas the FD was remarkably lower for XBC with 9.0 ± 0.2 g/100 cm^2^ (Figure 6). The difference in FD between the BC dressings is probably partly due to the differing overall water content (BC_A > 98%, XBC > 96%). The gel dressing Suprasorb^®^ G (GEL) consists of an acrylic polymer-based hydrogel with a water content of ~70% [76] and consequently showed the lowest FD of all tested samples with 2.0 ± 0.9 g/100 cm^2^. Considering the experimental dressing samples BC_C1 and BC_C2, the reduced water content of BC_C2 also correlates with a low FD capability. It stands out, however, that the modification in the case of BC_C1 did not lead to a reduction in the FD; the dressing performed similarly to BC_A in the experiment. Although this outcome seems contradictory at first, a possible rationalization could be that BC_C1 features not only an increased solid content but also a higher overall mass per area, whereas the absolute amount of water is comparable to that of BC_A. Taken together, these findings suggest that BC_C1 is as equally suited as the commercial dressing BC_A for the external hydration of a dry resp. low-exuding wound maintaining a suitable moist environment for successful wound healing, whereas the higher absorption capacity also suggests application on medium- to higher-exudation wounds.

### 2.7. Overview of Results

The results of all performed in vitro tests are shown in Table 1.

## 3. Materials and Methods

### 3.1. Post-Market Clinical Follow-Up Study Design

Commercially available BC wound dressing epicite^hydro^ (BC_A) was evaluated in a multi-center study in 44 patients with mainly venous leg ulcers, mixed leg ulcers, and diabetic foot syndrome. This observational data collection has been performed according to Medical Devices Documents (MEDDEV) 2.12/2 Rev. 2 guidelines for post-market clinical follow-up studies in compliance with German medical devices legislation (§§ 20 ff. Medical Device Act). Therefore, formal ethical approval was not required.

The dressing was evaluated regarding criteria such as wound area and depth progression, fibrous tissue, exudation, hydration, and state of wound margins, among others. The baseline of the study is shown in Table 2, whereas diagnoses, previous local treatment, and causal therapy are provided as Supplementary Materials (Tables S2–S4).

Wound area determination was carried out by either measurement of wound length and width or calculation by wound-documentation software. Fibrous tissue share was assessed by visual inspection of wound surface. Exudation and wound hydration were evaluated through interviews with participating physicians and care specialists. To assess condition of wound margins, one or several of the following parameters: ‘free from irritations, reddened, edematous, macerated, discolorated skin, necrotic, keratotic or undermined conditions’, were chosen. Statistical analysis was performed using SPSS.

### 3.2. In Vitro Tests

#### 3.2.1. Materials

The commercially available wound dressings tested in in vitro tests included BC dressing epicite^hydro^ (QRSKIN GmbH, Würzburg, Germany) (BC_A), bacterial cellulose dressing Suprasorb^®^ X (XBC), calcium alginate dressing Suprasorb^®^ A (ALG), and gel dressing Suprasorb^®^ G (GEL), all by Lohmann & Rauscher GmbH & Co. KG (Neuwied, Germany); polyurethane foam dressing ALLEVYN Gentle (FOAM_1) by Smith & Nephew Medical Ltd. (Hull, UK); soft silicone foam dressing Mepilex^®^ (FOAM_2) by Mölnlycke Health Care AB (Göteborg, Sweden); hydrocolloid dressing (HC) Hydrocoll^®^ (PAUL HARTMANN Limited, Heywood, UK); and sodium carboxymethylcellulose dressing Aquacel^®^ Extra™ (CMC) by ConvaTec Limited (Flintshire, UK). Sodium chloride was purchased from VWR Chemicals (Radnor, PA, USA). Calcium chloride dihydrate was obtained from Merck KgaA (Darmstadt, Germany) and gelatin 180 bloom was purchased from Carl Roth GmbH + Co KG (Karlsruhe, Germany).

#### 3.2.2. Preparation of Native BC

Experimental, non-commercial BC dressings (BC_C1 and BC_C2) were produced using the *Komagataeibacter xylinus* strain DSM 14666, deposited at the German Collection of Microorganism and Cell Cultures (DSMZ, Braunschweig, Germany) in a static cultivation technique as previously described [36,37]. Briefly, *K. xylinus* was cultivated at 28 °C in a process-controlled pilot plant with an area of 1 m^2^ (JeNaCell GmbH, Jena, Germany) using Hestrin–Schramm culture medium (HSM) [77]. In order to obtain BC dressings with high cellulose content, cultivation time and volume of HSM were adjusted empirically, so that a thickness of 8 mm was accomplished, which was chosen in order to double the thickness of most commercially available BC-based wound dressings (4 mm). Following cultivation, harvesting, and purification, BC dressings were cut into square pieces of 5 cm × 5 cm, sterilized by autoclaving (121 °C, 20 min, 2 bar), and stored at room temperature until further tests were carried out. Moisture content of BC_C was modified by flattening out the never-dried dressings by homogeneous vertical pressure until the sample weight reached an empirically predetermined weight range. BC_C1 and BC_C2 were produced with a weight range of 6.3 to 7.0 g per 5 cm × 5 cm sample, resp. 1.3 to 2.0 g per 5 cm × 5 cm sample.

#### 3.2.3. Solid Content

Solid Content (SC) was evaluated in accordance with Beekmann et al. [37]. Briefly, moisture-adjusted dressings were cut into pieces of approximately 1 cm^2^ and weighed (initial mass; m_i_). After air-drying for 48 h at room temperature (RT), weighing was repeated (dry mass; m_d_). SC was subsequently calculated using Equation (1):

(1) $$\mathrm{SC}\;\left[\%\right]=\frac{m_{d}}{m_{i}}\times 100\%$$

#### 3.2.4. Free Swell Absorptive Capacity

In order to assess the capability of different wound dressing materials to absorb fluid from heavily exuding wounds, a technique based on EN 13726-1:2002 was used. Minor modifications were made to align the method with the ongoing revision published as draft prEN 13726:2021-02 [78,79]. In short, 5 cm × 5 cm sized wound dressings were measured to the nearest 0.1 cm to determine the exact sample area (A) and weighed in a dry state (m_0_). Wound exudate was simulated by a test solution containing 142 mmol sodium and 2.5 mmol calcium as chloride salts (Test Solution A). Dressings were placed in a petri dish and immersed completely in the prewarmed test solution (37 °C ± 2 °C), transferred to an incubator (Binder GmbH, Tuttlingen, Germany), and allowed to soak for 0.5 h, 2 h, and 24 h ± 1 min at 37 °C ± 2 °C. At the end of each period, samples were removed from the test solution and excess fluid was allowed to drip off for 30 s ± 5 s. The dressings were reweighed (m_0.5 h_, m_2 h_, m_24 h_) and transferred back to the test solution for the remaining time until the test was completed. Each test was performed on 5 samples. Absorptive capacity (AC) was calculated for each test duration using Equation (2):

(2) $${\mathrm{AC}}_{t}\;\left[\frac{g}{100{\;\mathrm{cm}}^{2}}\right]=\frac{\left(m_{t}-m_{0}\right)\times 100}{A}\;\;t\;=\;\mathrm{test}\;\mathrm{duration}\;=0.5\;h\;\left|\;2\;h\;\right|\;24\;h$$

#### 3.2.5. Absorptive Capacity under Pressure

To assess the absorptive capacity of dressing materials under pressure, the investigation was carried out in accordance with the method proposed in prEN 13726:2021 with minor modifications [78]. Briefly, the setup described in Section 3.2.4 was adapted by placing the wound dressings on a perforated plate before immersing them in Test Solution A. Once the dressing was completely submerged, a pressure of 40 mmHg was applied using a weight of 1359.5 g with the help of a rigid compression plate between the weight and the dressing for homogenous pressure distribution. After 0.5 h, the setup consisting of a perforated plate, dressing, compression plate, and weight was removed from the test solution and allowed to drain for 5 min ± 10 s while pressure was still applied. Finally, the dressing was reweighed (m_0.5 h_) and AC was calculated as reported above.

#### 3.2.6. Moisture Vapor Transmission Rate

**Figure 7:**
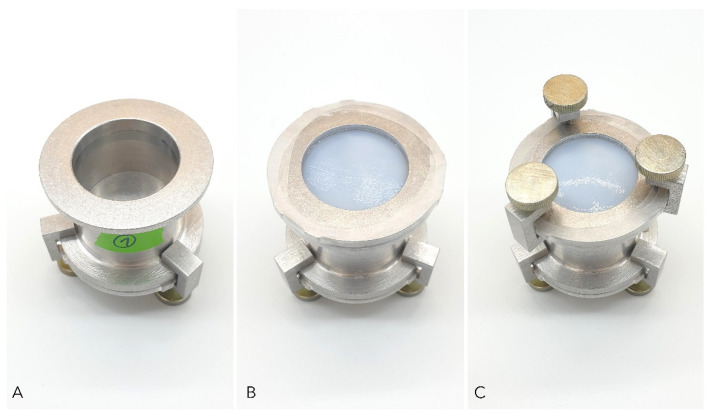
Standard Paddington cup method used to determine moisture vapor transmission rate (MVTR) of different wound dressings. (**A**) Paddington cup with defined orifice of 10 cm^2^ filled with deionized water; (**B**) Dressing sample placed over the orifice secured with clamping plate and sealed with impermeable film to prevent evaporation through the edges; (**C**) Final setup for measuring MVTR; fixation of the setup with screw clamps.

Moisture vapor transmission rate (MVTR) was evaluated according to EN 13726-2:2002. For ALG, CMC, HC, XBC, FOAM_1, FOAM_2, BC_A, BC_C1, and BC_C2 test variation A, ‘MVTR in contact with *vapor’* was carried out. Briefly, the dry dressing materials were clamped into the upper part of a standardized Paddington cup (test area: 10 cm^2^, The Surgical Materials Testing Laboratory, Bridgend, UK) filled with deionized water leaving a 5 mm gap between the water surface and the dressing. Evaporation through the dressing at 37 °C and <20% relative humidity (RH) was measured by weighing the cup at the beginning (m_1_) and the end (m_2_) of the 18 h to 24 h testing period (Figure 7).

For HC, XBC, FOAM_1, FOAM_2, BC_A, BC_C1, and BC_C2 test variation B, ‘MVTR in contact with *liquid’* was assessed as proposed in EN 13726-2:2002 with minor modifications. The upper part of the Paddington cup was covered with the dressing as described above. Afterward, the cup was inverted, filled with 20 mL of deionized water, and incubated at 37 °C, <20% RH for 18 h to 24 h. Determination of evaporation by weight loss was executed as described above. The Paddington cup was modified using a base plate with a fine vent hole (0.25 mm) on the opposite side of the dressing to prevent stretching of the dressing as a consequence of negative pressure inside the cup. All experiments were performed fivefold and MVTR for each variation was calculated using Equation (3):

(3) $$\mathrm{MVTR}\;\left[\frac{g}{m^{2}\times 24\;h}\right]=\left(m_{1}-m_{2}\right)\times 1000\times \frac{24}{T}\;\;m_{1}=\mathrm{weight}\;\mathrm{before}\;\mathrm{incubation}\;\left[g\right]\;m_{2}=\mathrm{weight}\;\mathrm{after}\;\mathrm{incubation}\;\left[g\right]\;T=\mathrm{testing}\;\mathrm{duration}\;\left[h\right]$$

#### 3.2.7. Fluid Donation

**Figure 8:**
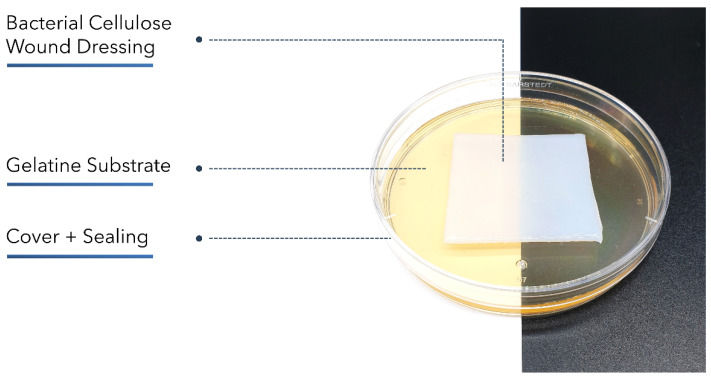
Setup based on EN 13726-1:2002 part 3.4 to compare moisture-donating wound dressings regarding their fluid donation. A gelatin gel with a water content < 35% was weighed before and after 48 h of contact with the dressing samples. The petri dish was covered and sealed tightly to prevent evaporation.

The wet dressings XBC, BC_A, BC_C, and GEL were compared regarding their fluid donation abilities using a method based on EN 13726-1:2002 part 3.4. Briefly, 35.00 g of gelatin powder was suspended in 65.00 g Test Solution A and incubated for 12 h at 60 °C until completely dissolved. An amount of 30 g of gelatin solution was poured into a petri dish of 90 mm diameter. The petri dishes were closed, sealed tightly, and incubated for 3 h at 25 °C to allow gel-forming (Figure 8).

Petri dishes were opened and weighed (m_1_), and 5 cm × 5 cm sized wound dressings were placed on the gel surface. After closing and sealing, the dishes were incubated vertically at 25 °C for 48 h. Lid was removed and petri dishes were weighed again after removal of the dressing samples (m_2_). Fluid Donation (FD) was calculated using Equation (4):

(4) $$\mathrm{FD}\;\left[\frac{g}{100{\;\mathrm{cm}}^{2}}\right]=\frac{\left(m_{2}-m_{1}\right)\times 100}{A}\;\;m_{1}=\mathrm{weight}\;\mathrm{before}\;\mathrm{incubation}\;\left[g\right]\;m_{2}=\mathrm{weight}\;\mathrm{after}\;\mathrm{incubation}\;\left[g\right]$$

## 4. Conclusions

Millions of patients worldwide suffer from chronic wounds. Although there are numerous types of wound dressings available in the market, selecting a suitable dressing can be demanding and therapy success is not self-evident. Wound dressings based on BC such as epicite^hydro^ have gained increasing relevance and show excellent results in the treatment of burn wounds. The post-market surveillance study featuring epicite^hydro^ presented herein revealed promising results regarding wound cleansing, pain reduction, a reduction in the wound area, and depths as well as fibrous tissue in chronic wounds. Nonetheless, there was still room for improvement in exudate management, where in vitro tests revealed that epicite^hydro^ fell short of higher absorptive materials such as foam dressings, alginates, or hydrofibers in terms of exudate absorption.

In this work, the exudate-handling properties of BC dressings could be altered using a readily feasiblemodification to provide a remarkably higher absorption capacity, which represents a promising enhancement concerning the therapy of medium- to higher-exuding wounds. For the first time, a potential interplay between the MVTR in contact with liquid and the free swell absorptive capacity was described, the latter being surprisingly higher for BC dressings featuring a moisture content of 92% or 97%, respectively, than for dry, advanced wound dressings made of alginates, carboxymethylcellulose (hydrofibers), or hydrocolloids. This unique property could be explained by capillary forces within the open-porous and fine network structure of BC dressings. Both observations, the increase in the free swell absorptive capacity over time (here: within 24 h), and the significantly lower free swell absorptive capacity of the moisture-saturated version (BC_A) support this hypothesis. Future studies should investigate whether these results can be replicated in alternative experimental settings and could be effective in clinical use. Biomimetic concepts such as wound-healing (scratch) assays, which have already been successfully applied to BC-based wound dressings, could also be helpful in this context [36,80].

At the same time, the proven beneficial characteristics, such as a suitable permeability and a strong ability to donate moisture, which have a clinically relevant impact on the hydrobalance of wounds, could be maintained in the new versions BC_C1 and BC_C2. Carbohydrate polymer-based wound dressings made of bacterial cellulose such as epicite^hydro^ thus represent a very promising and adaptable material for the successful treatment of both burn and chronic wounds of different origins.

## Figures and Tables

**Table 1:** Overview of evaluated parameters, tests, and results.

Wound Dressing	Solid Content	Free Swell Absorptive Capacity (0.5 h; 2 h; 24 h)	Absorptive Capacity under Pressure	MVTR In Contact With Vapor	MVTR in Contact with Liquid	Fluid Donation to Gelatin
Unit	%	g/100 cm^2^	g/100 cm^2^	g/m^2^/24 h	g/m^2^/24 h	g/100 cm^2^
ALG	-	17.6 ± 1.3	11.0 ± 1.4	6135 ± 78	-	-
		18.0 ± 1.1				
		18.4 ± 1.1				
CMC	-	20.1 ± 1.7	17.2 ± 0.5	6844 ± 282	-	-
		20.3 ± 1.7				
		21.0 ± 1.3				
HC	-	3.9 ± 0.3	3.0 ± 0.9	98 ± 5	330 ± 42	-
		6.0 ± 0.4				
		15.2 ± 1.1				
FOAM_1	-	82.0 ± 2.9	35.3 ± 2.5	3510 ± 225	12,790 ± 747	-
		85.1 ± 5.4				
		92.5 ± 6.4				
FOAM_2	-	60.1 ± 1.6	32.0 ± 1.9	2808 ± 103	16,750 ± 419	-
		58.6 ± 3.9				
		61.1 ± 3.4				
XBC	3.16 ± 0.18	13.3 ± 0.5	2.2 ± 0.3	3458 ± 198	27,663 ± 959	9.0 ± 0.2
		18.4 ± 0.9				
		22.1 ± 1.2				
BC_A	1.57 ± 0.10	6.7 ± 0.5	−2.9 ± 2.2	4039 ± 230	27,648 ± 488	18.9 ± 1.8
		8.7 ± 0.8				
		9.9 ± 0.9				
BC_C1	3.27 ± 0.27	23.2 ± 5.1	1.7 ± 0.5	2697 ± 118	26,334 ± 1465	19.1 ± 1.7
		31.2 ± 5.6				
		36.1 ± 5.7				
BC_C2	8.20 ± 0.57	30.1 ± 6.3	13.1 ± 1.5	2908 ± 95	27,212 ± 913	3.0 ± 0.3
		37.4 ± 6.5				
		43.9 ± 7.7				
GEL	-	-	-	-	-	2.0 ± 0.9

**Table 2:** Baseline of BC_A Post-Market Clinical Follow-up study.

	**Male**	**Female**	
Sex	22	22	
	**Mean**	**Median**	**SD**
Patient Age [years]	66.9	71	15.94
Wound Age [weeks]	66.56	13	120.04
	**Yes**	**Not specified**	
Diagnosis of underlying disease	42	2	
Treatment of underlying disease	29	15	

## Data Availability

The data contained within the article and supplementary.

## Supplementary Materials

The following are available online at https://www.mdpi.com/article/10.3390/ph15060683/s1, Table S1: Clinical trials assessing different BC-based wound dressings in chronic wound care. Table S2: Clinical trials assessing different BC-based wound dressings in burn wound care. Table S3: Overview of wound diagnoses in BC_A Post-Market Clinical Follow-up Study. Table S4: Overview of local wound treatment prior to BC_A Post-Market Clinical Follow-up Study. Table S5: Overview of causal therapy in BC_A Post-Market Clinical Follow-up Study. Figure S1: Moisture vapor transmission rate (MVTR) of different wound dressings determined with standard Paddington cup in contact with vapor method (mean ± SD; *n* = 5). References [81,82,83,84,85,86,87,88,89,90,91] are cited in the supplementary materials.

## Abbreviations & Terms

AC	Absorptive Capacity
ALG	Calcium Alginate dressing Suprasorb^®^ A (Lohmann & Rauscher GmbH & Co. KG, Neuwied, Germany)
BC	Bacterial Cellulose
BC_A	BC based wound dressing epicite^hydro^ (QRSKIN GmbH, Würzburg, Germany)
BC_C1	Experimental, non-commercial and modified BC-based wound dressing
BC_C2	Experimental, non-commercial, and modified BC Cellulose-based wound dressing
CCl	Compression Class
CMC	Sodium carboxymethylcellulose dressing Aquacel^®^ Extra™ (ConvaTec Limited, Flintshire, UK)
DSMZ	Deutsche Sammlung von Mikroorganismen und Zellkulturen (German Collection of Microorganisms and Cell Cultures)
FD	Fluid Donation
FOAM_1	Polyurethan foam dressing ALLEVYN Gentle (Smith & Nephew Medical Ltd., Hull, UK)
FOAM_2	Soft silicone foam dressing Mepilex^®^ (Mölnlycke Health Care AB, Göteborg, Sweden)
GEL	Gel dressing Suprasorb^®^ G (Lohmann & Rauscher GmbH & Co. KG, Neuwied, Germany)
HC	Hydrocolloid dressing Hydrocoll^®^ (PAUL HARTMANN Limited, Heywood, UK)
HET-CAV	Shell-less Hen’s Egg Test on Chick Area Vasculosa
HSM	Hestrin–Schramm culture medium
*K. xylinus*	*Komagataeibacter xylinus*
MTT	3-(4,5-dimethylthiazol-2-yl)-2,5-diphenyltetrazolium bromide
MVTR	Moisture Vapor Transmission Rate
PMCF	Post-Market Clinical Follow-up (Study)
SC	Solid Content
VLU	Venous Leg Ulcer
XBC	BC-based wound dressing Suprasorb^®^ X (Lohmann & Rauscher GmbH & Co. KG, Neuwied, Germany)
