# Supplementary material for: Bacterial Cellulose—Adaptation of a Nature-Identical Material to the Needs of Advanced Chronic Wound Care
Source: Pharmaceuticals (Basel). 2022 May 30;15(6):683. doi: 10.3390/ph15060683 (PMC9228795; doi:10.3390/ph15060683)
Supplement: Supplementary file 1 [file pharmaceuticals-15-00683-s001.zip › pharmaceuticals-1706803-supplementary.pdf]

pharmaceuticals

MDPI

Supplementary Information: Adaptation of a Nature-Identical Material to the Needs of Advanced Chronic Wound Care

Paul Zahel [1,2], Uwe Beekmann [1], Thomas Eberlein [3], Michael Schmitz [4], Oliver Werz [2] and Dana Kralisch [1,5*]

[1] JeNaCell GmbH – An Evonik Company, 07745 Jena, Germany; paul.zahel@evonik.com (P.Z.); uwe.beekmann@evonik.com (U.B.); dana.kralisch@evonik.com (D.K.)
[2] Department of Pharmaceutical/Medicinal Chemistry, Institute of Pharmacy, Friedrich Schiller University Jena, 07743 Jena, Germany; paul.zahel@uni-jena.de (P.Z.); oliver.werz@uni-jena.de (O.W.)
[3] Akademie-ZWM AG, 8424 Embrach, Switzerland; thomaseberlein@hotmail.com (T.E.)
[4] MCS Medical Consulting, 56414 Oberahr, Germany; schmitzmichael@mcsoberahr.onmicrosoft.com
[5] Evonik Operations GmbH, 45128 Essen, Germany
* Correspondence: dana.kralisch@evonik.com

Table S1. Clinical trials assessing different BC-based wound dressings in chronic wound care.

Author	Indication	Study design	Patients	Treatment	Control	Conclusion
Alvarez et al. [1]	Venous leg ulcers	Prospective RCT	24 adults	Wet BC-dressing (XCell®) + compression	Nonadherent silicone dressing + compression	More effective autolytic debridement; reduced time to granulation; greater wound area reduction; less wound pain
Portal et al. [2]	Nonhealing lower extremity ulcerss	Retrospective observational study	11 adults	Dry BC-dressing (Dermafill™)	Various standard of care products	Shortened time to wound closure over standard care
Solway et al. [3]	Diabetic foot ulcers	Prospective RCT	30 adults	Dry BC-dressing (Dermafill™)	Petrolatum gauze	Faster wound closure rate
Dini et al. [4]	Venous leg ulcers	Prospective RCT	46 adults	Wet BC-dressing (Suprasorb® X) + foam + compression	Foam + compression	Improved leg ulcer healing and restoration of skin barrier function
Cavalcanti et al. [5]	Venous leg ulcers	Prospective RCT	25 adults	Wet non-commercial BC-dressing + gauze	Medium chain triglycerides oil + gauze	Decreased pain; earlier discontinuation of analgesic use
Colenci et al. [6]	Venous leg ulcers	Prospective RCT	46 adults	Dry BC-dressing (Nanoskin®) + compressive elastic band	Collagenase dressing + compressive elastic band	Similar results in both groups regarding pain, quality of life, safety; increased vascularization and promoted early wound healing for BC
Maia et al. [7]	Lower limb revascularization of ischaemic wounds	Prospective RCT	24 adults	BC-dressing + gel	Essential fatty acids + gauze	Faster wound area reduction, higher complete healing rate after 90 days

Table S2. Clinical trials assessing different BC-based wound dressings in burn wound care.

Author	Indication	Study design	Patients	Treatment	Control	Conclusion
Piatkowski et al. [8]	Second-degree burns	Prospective RCT	60 adults	Wet polyhexanide-loaded BC-dressing (Suprasorb® X)	Silver sulphadiazine cream	Better and faster pain reduction, cost-effective and safe application of BC-dressing
Aboelnaga et al. [9]	Second-degree burns	Prospective RCT	40 adults	Wet BC-dressing (EpiProtect®) + plastic film + elastic bandage	Silver sulphadiazine cream + gauze + elastic bandage	Shorter hospitalization length, lower pain score during and after wound care, fewer dressing changes
delli Santi et al. [10]	Second/third-degree burn	Pilot study	5 children	Wet BC-dressing (EpiProtect®) after enzymatic debridement with Nexobrid™	-	BC-dressing effective to heal pediatric burn wounds after enzymatic debridement
Karlsson et al. [11]	First-degree and excised burns	Retrospective comparative case review	38 children & adults	Wet BC-dressing (EpiProtect®)	Porcine xenograft	Similar outcome for both groups, better pain relief for BC-dressing
Cattalaens et al. [12]	Second/third-degree burns	Retrospective evaluation	56 children	Wet BC-dressing (Epicite^{hydro}) + petrolatum gauze + dry compress	-	Easy applicability, rapid re-epithelialization, excellent wound hydration, no wound-associated infections, no hypertrophic scars, good wound healing
Shanks et al. [13]	First- to third degree burns	Prospective case series	30 children	Wet BC-dressing (EpiProtect®) + silicone gauze	-	Effective analgesia for dressing changes, safe, reliable and well-tolerated for pediatric patients
Maurer et al. [14]	First- to third degree burns	Retrospective comparative trial	190 children	Wet BC-dressing (Epicite^{hydro}) + non-adhesive gauze + dry compress	Polyurethane foam dressing	Similar for wound healing, rate of complications and skin grafting, shorter hospitalization length and less interventions with anesthesia for BC
Resch et al. [15]	Second-degree burns	Prospective case series	16 children	Wet BC-dressing (Epicite^{hydro}) + non-adhesive gauze + dry compress	-	Undisturbed recovery of burn, frequency of dressing changes up to seven days
Schiefer et al. [16]	Second-degree burns	Prospective open comparative trial	20 adults	Wet BC-dressing (Epicite^{hydro}) + non-adhesive gauze + external dressing	SUPRATHEL® skin substitute	Similar outcome for both groups with regard to pain reduction, bleeding, infection, exudation, and scarring, better cost-effectivity for BC-dressing

Table S3. Overview of wound diagnoses in BC_A Post-Market Clinical Follow-up Study.

Wound Diagnosis	Patient Count
Venous Leg Ulcer (VLU)	18
Mixed Leg Ulcer (MLU)	8
Diabetic Foot Syndrome (DFS)	4
MLU + DFS	1
Wound Dehiscence	1
Decubitus Ulcer, unspecified	2
Decubitus Ulcer Stage II	2
Decubitus Ulcer, not assessable	1
Charcot Foot, non-diabetic	1
Leg Ulcer, concomitant vasculitis	1
Acne inversa	1

Table S4. Overview of local wound treatment prior BC_A Post-Market Clinical Follow-up Study.

Previous Local Wound Treatment	Patient Count
Sodium Carboxymethylcellulose (Hydrofiber) Dressing	6
Sterile Gauze	2
Foam Dressing	3
Alginate Dressing	2
Hydrocolloid Dressing	1
Polyester Contact Mesh with Medical Honey (Vivamel® Contact)	2
Silver-coated antimicrobial Dressing (Acticoat® Flex 3)	1
Antimicrobial hydrogel-impregnated Acetate Dressing (Cutimed® Sorbact® Gel)	1
Silver-coated Alginate Dressing	3
Iodine Solution	1
Silver Sulphadiazine Cream (FLAMMAZINE®)	1
Silver-coated Sodium Carboxymethylcellulose Dressing (AQUACEL® AG)	4
Silver-coated Absorbent Activated Charcoal Dressing	4
Absorbent Activated Charcoal Dressing	1
ActiMaris® Wound Gel	1
Medical Honey impregnated Gauze	2
Hydrogel	2
Hydrophobic Wound Dressing	2
Various	1
Unspecified	4

Table S5. Overview of causal therapy in BC_A Post-Market Clinical Follow-up Study.

Causal Therapy	Patient Count
Compression Therapy	10
Revascularization/ Percutaneous Transluminal Angioplasty	2
Abdominal Binder	1
Regular Assessment of Ankle-Brachial Index	1
Pressure Relief	6
Supportive Orthosis	1
Pharmacotherapy: Prednisolone	1
Pharmacotherapy: Metformin	1
Vacopaso® Free Therapy Shoe	1

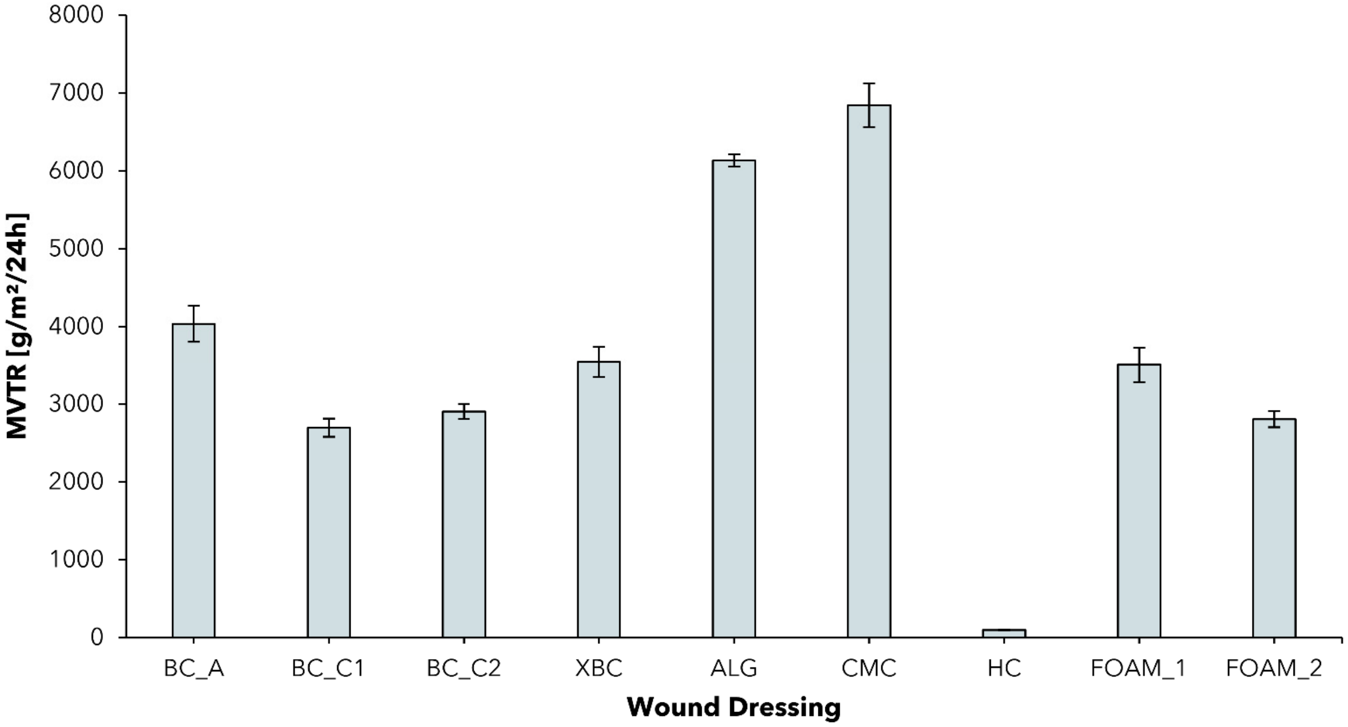

Figure S1. Moisture vapor transmission rate (MVTR) of different wound dressings determined with standard Paddington cup in contact with vapor method (mean ± SD; n = 5).

References

1. Alvarez, O.M.; Patel, M.; Booker, J.; Markowitz, L. Effectiveness of a Biocellulose Wound Dressing for the Treatment of Chronic Venous Leg Ulcers: Results of a Single Center Randomized Study Involving 24 Patients. *Wounds Compend. Clin. Res. Pract.* **2004**, *16*, 224–233.
2. Portal, O.; Clark, W.A.; Levinson, D.J. Microbial Cellulose Wound Dressing in the Treatment of Nonhealing Lower Extremity Ulcers. **2009**, 2.
3. Solway, D.R.; Clark, W.A.; Levinson, D.J. A Parallel Open-Label Trial to Evaluate Microbial Cellulose Wound Dressing in the Treatment of Diabetic Foot Ulcers. *Int. Wound J.* **2011**, *8*, 69–73, doi:10.1111/j.1742-481X.2010.00750.x.
4. Dini, V.; Romanelli, M.; Andriessen, A.; Barbanera, S.; Bertone, M.S.; Brilli, C.; Abel, M. Improvement of Periulcer Skin Condition in Venous Leg Ulcer Patients: Prospective, Randomized, Controlled, Single-Blinded Clinical Trial Comparing a Biosynthetic Cellulose Dressing with a Foam Dressing. **2013**, *26*, 8.

5. Cavalcanti, L.M.; Pinto, F.C.M.; Oliveira, G.M.D.; Lima, S.V.C.; Aguiar, J.L.D.A.; Lins, E.M. Efficacy of Bacterial Cellulose Membrane for the Treatment of Lower Limbs Chronic Varicose Ulcers: A Randomized and Controlled Trial. *Rev. Colégio Bras. Cir.* **2017**, *44*, 72–80, doi:10.1590/0100-69912017001011.
6. Colenci, R.; Miot, H.A.; Marques, M.E.A.; Schmitt, J.V.; Basmaji, P.; Jacinto, J. dos S.; Abbade, L.P.F. Cellulose Biomembrane versus Collagenase Dressing for the Treatment of Chronic Venous Ulcers: A Randomized, Controlled Clinical Trial. *Eur. J. Dermatol.* **2019**, *29*, 387–395, doi:10.1684/ejd.2019.3608.
7. Maia, A.L.; Lins, E.M.; Aguiar, J.L.A.; Pinto, F.C.M.; Rocha, F.A.; Batista, L.L.; Fernandes, W.R. de M.A. Curativo Com Filme e Gel de Biopolímero de Celulose Bacteriana No Tratamento de Feridas Isquêmicas Após Revascularização de Membros Inferiores. *Rev. Colégio Bras. Cir.* **2019**, *46*, e20192260, doi:10.1590/0100-6991e-20192260.
8. Piatkowski, A.; Drummer, N.; Andriessen, A.; Ulrich, D.; Pallua, N. Randomized Controlled Single Center Study Comparing a Polyhexanide Containing Bio-Cellulose Dressing with Silver Sulfadiazine Cream in Partial-Thickness Dermal Burns. *Burns* **2011**, *37*, 800–804, doi:10.1016/j.burns.2011.01.027.
9. Aboelnaga, A.; Elmasry, M.; Adly, O.A.; Elbadawy, M.A.; Abbas, A.H.; Abdelrahman, I.; Salah, O.; Steinvall, I. Microbial Cellulose Dressing Compared with Silver Sulphadiazine for the Treatment of Partial Thickness Burns: A Prospective, Randomised, Clinical Trial. *Burns* **2018**, *44*, 1982–1988, doi:10.1016/j.burns.2018.06.007.
10. delli Santi, G.; Borgognone, A. The Use of Epiprotect®, an Advanced Wound Dressing, to Heal Paediatric Patients with Burns: A Pilot Study. *Burns Open* **2019**, *3*, 103–107, doi:10.1016/j.burnso.2019.05.001.
11. Karlsson, M.; Olofsson, P.; Steinvall, I.; Sjöberg, F.; Thorfinn, J.; Elmasry, M. Three Years' Experience of a Novel Biosynthetic Cellulose Dressing in Burns. *Adv. Wound Care* **2019**, *8*, 71–76, doi:10.1089/wound.2018.0790.
12. Cattelaens, J.; Turco, L.; Berclaz, L.M.; Huelsse, B.; Hitzl, W.; Vollkommer, T.; Bodenschatz, K.J. The Impact of a Nanocellulose-Based Wound Dressing in the Management of Thermal Injuries in Children: Results of a Retrospective Evaluation. *Life* **2020**, *10*, 212, doi:10.3390/life10090212.
13. Shanks, L.A.; Cronshaw, A.; Alexander, K.S.; Davies, J.A.; O'Boyle, C.P. Evaluation of EpiProtect® Microbial Cellulose Burns Dressings in Young Children. *Scars Burns Heal.* **2020**, *6*, 205951312094050, doi:10.1177/2059513120940503.
14. Maurer, K.; Renkert, M.; Duis, M.; Weiss, C.; Wessel, L.M.; Lange, B. Application of Bacterial Nanocellulose-Based Wound Dressings in the Management of Thermal Injuries: Experience in 92 Children. *Burns* **2021**, S0305417921001728, doi:10.1016/j.burns.2021.07.002.
15. Resch, A.; Staud, C.; Radtke, C. Nanocellulose-based Wound Dressing for Conservative Wound Management in Children with Second-degree Burns. *Int. Wound J.* **2021**, *18*, 478–486, doi:10.1111/iwj.13548.
16. Schiefer, J.L.; Aretz, G.F.; Fuchs, P.C.; Bagheri, M.; Funk, M.; Schulz, A.; Daniels, M. Comparison of Wound Healing and Patient Comfort in Partial-thickness Burn Wounds Treated with SUPRATHEL and Epicte ^{hydro} Wound Dressings. *Int. Wound J.* **2021**, iwj.13674, doi:10.1111/iwj.13674.
